# Syntélencéphalie: variant inter hémisphérique médian de l´holoprosencéphalie

**DOI:** 10.11604/pamj.2022.42.193.33462

**Published:** 2022-07-08

**Authors:** Daoud Bentaleb, Siham Salam

**Affiliations:** 1 Service de Radiologie-Pédiatrique de l´Hôpital d´Enfants Abderrahim Harouchi, Centre Hospitalier Universitaire Ibn Rochd de Casablanca, Casablanca, Maroc

**Keywords:** Holoprosencéphalie, imagerie par résonnance magnétique, syntélencéphalie, Holoprosencephaly, Magnetic Resonance Imaging (MRI), syntelencephaly

## Abstract

Holoprosencephaly (HPE) is a congenital malformation occurring very early in pregnancy and due to abnormal diverticulation of the prosencephalon. This defect in primary brain cleavage may be total or partial, which explains the anatomopathological variants of holoprosencephaly. The main forms of holoprosencephaly include alobar, semilobar and lobar HPE. Syntelencephaly also known as middle interhemispheric variant (MIHV) is a very rare type of HPE. While in the three most common variants, cleavage defect has antero-posterior gradient of severity, syntelencephaly does not follow this rule. The latter is characterised by abnormal communication between cerebral hemispheres in the posterior and parietal frontal regions, with normal inter-hemispheric separation of the anterior frontal lobes, of the occipital regions and central grey nuclei. The trunk of the corpus callosum is achieved at the level of the knee of the corpus callosum (allowing differentiation from lobar HPE) and of the splenium. An azygos cerebral artery is common, consisting of a fusion of the A1 segments of the anterior cerebral arteries to form a single arterial trunk. Other cerebral malformations may be associated with syntelencephaly, the most frequent being cortical dysplasia and grey matter heterotopia. Facial abnormalities are less common in syntelencephaly compared to classic forms of HPE. Prognosis depends mainly on these malformative associations in the brain and other organs, and even on chromosomal aberrations.

## Image en médecine

L´holoprosencéphalie (HPE) est une malformation congénitale faisant partie des malformations cérébrales précoces dues à une anomalie de la diverticulation du prosencéphale. Ce défaut de clivage du cerveau primitif peut être total ou partiel, ce qui explique les variantes anatomopathologiques de l´holoprosencéphalie. Les principales formes d´holoprosencéphalie sont représentées par l´HPE alobaire, semilobaire et lobaire. La syntélencéphalie appelée également « variant inter hémisphérique médian de l'holoprosencéphalie » est une variante très rare de l´HPE. Si dans les trois variantes les plus communes, le gradient de gravité du « défaut de clivage » est antéro-postérieur, la syntélencéphalie n´obéit pas à cette règle. Cette dernière se caractérise par une communication anormale des hémisphères cérébraux dans les régions frontales postérieures et pariétales, avec une séparation inter hémisphérique normale des lobes frontaux antérieurs, des régions occipitales et des noyaux gris centraux (NGC). Le tronc du corps calleux est atteint avec un respect du genou, du corps (ce qui permet la différenciation avec l´HPE lobaire) et du splénium. Une artère cérébrale azygos est fréquente, elle consiste en une fusion des segments A1 des artères cérébrales antérieurs pour former un seul tronc artériel. D´autres malformations cérébrales peuvent s´associer à la syntélencéphalie, les plus fréquentes sont les dysplasies corticales et les hétérotopies de la substance grise. Les anomalies faciales sont moins fréquentes dans la syntélencéphalie par rapport aux formes classiques d´HPE. Le pronostic dépend essentiellement de ces associations malformatives cérébrales et des autres organes et également des aberrations chromosomiques.

**Figure 1:**
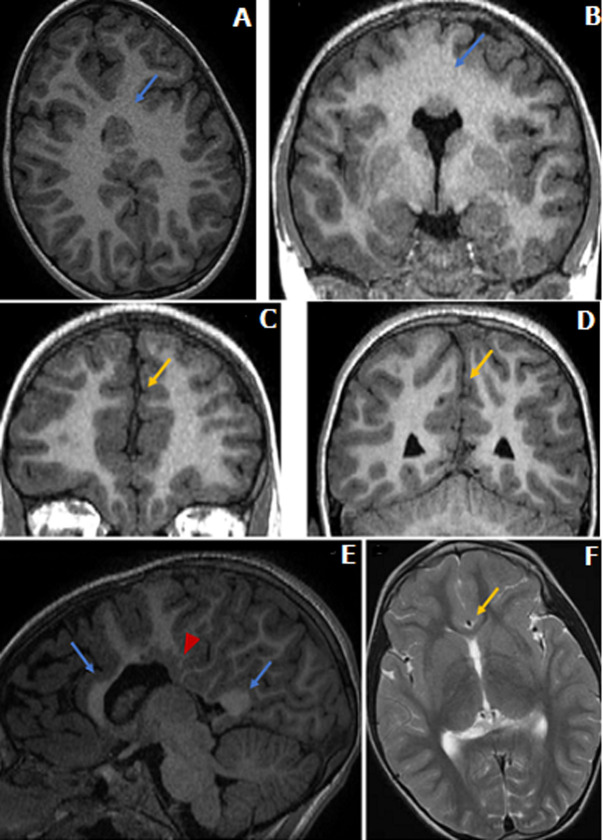
séquence axiale T1 (A), séquence coronale T1 (B,C,D); (A et B) fusion de la partie postérieure des lobes frontaux et des lobes pariétaux (flèche); (C) séparation normale des parties antérieures des lobes frontaux; (D) séparation normale des lobes occipitaux; IRM cérébrale avec une séquence sagittale T1 (E) et axiale T2 (F); (E) agénésie du tronc du corps calleux (tête de flèche) et aspect normal du genou et du splénium (flèches); (F) artère cérébrale antérieure azygos (flèche), à noter la séparation normale des noyaux gris centraux

